# Units of Remedial Agencies

**Published:** 1899-12

**Authors:** 


**A Monthly Review of Medicine and Surgery.**

*EDITOR:*

WILLIAM WARREN POTTER, M. D.

All communications, whether of a literary or business nature, books for review and exchanges should be addressed to the editor: 284 Franklin Street, Buffalo, N. Y.

Vol. XXXIX.—LV. DECEMBER, 1899. No. 5.

### UNITS OF REMEDIAL AGENCIES.

IN ALL physical sciences the foundation is considered to be laid when the standard units have been established. In pure chemistry that includes the determination of specific gravities, atomic weights and valences, it is recognised that these are essential prerequisites to all further study. Yet physiological sciences, the materia medica and therapeutics, have long been content to continue without any such definite fixation of the units. It was only ten years ago that the United States Pharmacopeia recognised the principle of standardisation when it admitted its application to opium, nux vomica and cinchona.

Certain drugs are easily reducible to such standardisation by chemical analysis of their constituent elements. The principle already recognised in the case of the three drugs, whose standardisation has been officially sanctioned in the present pharmacopeia, may readily be extended to others in the forthcoming revision. There is every reason to expect that the committee on revision will sanction the standardisation of calabar, hyoscyamus, colchicum, ipecac, veratrum, belladonna, gelsemium, podophyllum, conium and stramonium which are placed in the same category as opium and nux vomica by reason of their toxicity and their importance. No difficulty will be encountered in the extension of standardisation to such galenicals as may be tested by chemical assay, and the quantitative determination of their active constituents.

Yet there are other drugs of equal importance and of the same or even greater degree of toxic power which should be brought to a working standard just as much as those already mentioned. In the case of these, the ordinary methods of chemical assay are found to be inapplicable. Yet not on that account is their standardisation to be held impracticable. At least one of the great manufacturing establishments, which has devoted great attention to the quality of its pharmaceutical preparations, has carried the standardisation of these articles beyond the mere process of experiment and has reached successful results. In this method the test is physiological. The symptomatic effects of the drugs is comparatively illustrated on small animals, the frog having been found in practice the most readily available. The method of this physiological assay is remarkably simple, yet none the less effective. Frogs are treated with the drug of a certain recognised standard of potency, the object being to establish for the group of animals which they represent the minimum fatal dose. Upon other animals of the group the drug to be tested is tried experimentally for a similar determination of the minimum fatal dose. The ratio between the two doses thus ascertained will afford the percentage of the strength of the drug under test as compared with the standard. It has been the practice of the house which has made the greatest strides in the direction of this physiological assay to reject all the drugs which are found below the standard, a policy which we learn has been responsible for the rejection of many tons of crude drugs every year.

The revisers of the pharmacopeia need have no fear of the physiological assay as either a new or an unaccepted practice. It has been established in the case of several remedial agents with unfailing success, and the principle has been abundantly passed on in the case of antidiphtheritic serum. All that is asked of the revision committee is that they accept an accomplished fact and, by the weight of their approval sanction, make official a condition already in successful existence.

We have refrained from presenting more than the principle involved, for we are of opinion that the establishment of the principle as official is the best thing for modern medicine in the revised pharmacopeia. Yet the vital importance of the subject is to be seen in the most cursory examination of the drugs which will first be standardised under this process. These are cannabis indica, digitalis, aconite, ergot, and strophanthus. Just how important this standardisation is in the case of these drugs unamenable to chemical assay is exhibited in the case of strophanthus. Its active principle, strophanthin, is three times as poisonous as atropin, ten times as poisonous as strychnin, and twelve times as poisonous as absolute hydrocyanic acid. This shows the danger in using such a drug when the toxic qualities are not officially determined, and that danger constitutes in itself the best argument for the sanctioning of the physiological test, since in the case of the glucosides that is the only test which is available.

